# The Type of Dietary Fat in an Isocaloric Breakfast Meal Does Not Modify Postprandial Metabolism in Overweight/Obese Pregnant Women

**DOI:** 10.3390/nu11030490

**Published:** 2019-02-26

**Authors:** Mary N. R. Lesser, Kasuen Mauldin, Lisa Sawrey-Kubicek, Virginia Gildengorin, Janet C. King

**Affiliations:** 1 Children’s Hospital Oakland Research Institute, 5700 Martin Luther King Jr. Way, Oakland, CA 94609, USA; mnrlesser@berkeley.edu (M.N.R.L.); ggildengorin@mail.cho.org (V.G.); 2 Department of Nutritional Sciences & Toxicology, 119 Morgan Hall, University of California, Berkeley, CA 94720, USA; 3 Department of Nutrition, Food Science & Packaging, San Jose State University, One Washington Square, San Jose, CA 95192, USA; kasuen.mauldin@sjsu.edu; 4 Department of Nutrition, University of California, Davis, One Shields Avenue, Davis, CA 95616, USA; lsawreykubicek@ucdavis.edu

**Keywords:** metabolism, overweight, pregnancy, dairy, nuts

## Abstract

Almonds provide a satiating, healthy source of fat and fiber. The postprandial metabolic and satiety response to 2 ounces of nuts or dairy was assessed in 18 overweight/obese women during late pregnancy. Serum glucose, triglycerides, insulin, c-peptide, leptin, ghrelin, and lipoprotein particles were measured prior to and during a 5-h postprandial period following the consumption of an isocaloric breakfast meal with equivalent amounts of fat from either nuts or dairy on two separate mornings. Satiety was assessed by visual analogue scale (VAS) questionnaires and ad libitum food intake at the end of the study. At 33 weeks gestation, the women had gained an average of 7.0 ± 4.4 kg during gestation. Body fat averaged 41.9 ± 5.5% and hemoglobin A1c levels were elevated, (7.2 ± 0.6%). Fasting glucose levels were normal, but hyperinsulinemia was evident. The two test meals did not affect the postprandial metabolic response, but glucose, triglyceride, and ghrelin concentrations changed with time during the postprandial period (*p* < 0.001, *p* = 0.0008, *p* = 0.006). Satiety measures did not differ between the two test meals. Consuming an isocaloric breakfast meal with equivalent amounts of fat from nuts or dairy did not alter postprandial levels of blood lipids, glucose, hormones, or measures of satiety in overweight/obese, pregnant women.

## 1. Introduction

Half of all American women of childbearing age are overweight, and one-third are obese [1]. The prevalence of metabolic disorders during and after pregnancy has increased concurrently with the rise in maternal obesity [2,3,4]. Metabolic abnormalities (i.e., hyperglycemia) during pregnancy may predispose the offspring to subsequent obesity and chronic diseases [5,6,7]. Excessive weight gain during pregnancy by overweight and obese women creates a higher risk of prenatal complications, retention of excess body fat postpartum, and the development of metabolic diseases in the offspring later in life [8,9,10].

Overweight/obese, pregnant women are at increased risk for developing gestational diabetes and dyslipidemia [10]. The maternal insulin resistance that normally develops during pregnancy increases the placental uptake of glucose and lipid and the transfer of these substrates to the fetus, thus supporting fetal growth. Maternal obesity enhances the insulin resistance present during pregnancy and increases the risk of hyperglycemia and hypertriglyceridemia in late pregnancy [11]. Unhealthy eating patterns and suboptimal levels of physical activity contribute to excessive weight gain during pregnancy [11,12]. Thus, to improve pregnancy outcomes and the long-term health of the mother and child, clinical programs designed to control weight gain and reduce metabolic disorders are needed. Although dietary interventions are used routinely to reduce metabolic disease in non-pregnant obese individuals, no national or international dietary standards have been established for obese, pregnant women unless they develop Gestational Diabetes Mellitus (GDM) [13]. It is important, therefore, to identify healthy food sources that reduce the risk of glucose intolerance and dyslipidemia while increasing satiety. These foods could then be part of a recommended dietary pattern for overweight/obese pregnant women to support a healthy metabolism throughout pregnancy.

The consumption of unsaturated fatty acid-rich lipid food sources has been shown to improve cardiometabolic health. Almonds, which are high in monounsaturated fatty acids and fiber, provide flavor and satiety to the diet. Also, their net metabolizable energy is low, which helps manage body weight [14,15]. In non-pregnant adults, almond consumption reduced oxidative stress and inflammation and promoted vascular health and glycemic control [16,17,18]. The purpose of this study, therefore, was to compare the effects of two isocaloric fat sources, dairy (cream cheese) or nuts (almonds), on postprandial metabolism and satiety in overweight/obese pregnant women. Since almonds provide more unsaturated fat than cream cheese, we hypothesized that the postprandial metabolic response to almonds would be more favorable than the response to higher saturated fat, cream cheese.

## 2. Materials and Methods

### 2.1. Experimental Design

Eighteen overweight/obese, pregnant women in the third trimester were recruited to participate in a cross-over, randomized controlled trial examining the metabolic response to consuming equivalent amounts of dairy (cream cheese) or nuts (almonds) in two standardized, isocaloric breakfast meals matched for carbohydrate, protein, and fat levels. Clinical trial registration number NCT01919476. Each participant consumed both test meals on separate days within 3–7 days in random order (computer generated sequence). On the test days, the participants came to the Cholesterol Research Center (CRC) in Berkeley, CA, to complete the six-hour study. After a fasting blood sample was collected, the women consumed one of the test meals and postprandial blood samples were collected at 30 min, 1, 2, 3, 4, and 5 h to measure hormones and metabolic biomarkers of glucose and lipid metabolism. After completing the 5-h postprandial study, the women were offered an *ad libitum* lunch and the amount of food consumed was weighed and recorded.

### 2.2. Study Participants

Pregnant women were recruited from prenatal practices in Oakland/Berkeley, CA, to participate in the study. Inclusion criteria included being between 30–36 weeks of pregnancy, a pre-pregnancy BMI between 25–40 kg/m^2^, and a willingness to come to the CRC for two six-hour studies. Exclusion criteria included a diagnosis of gestational diabetes, diabetes pre-pregnancy, or other metabolic disorders (i.e., renal, thyroid, or bone disorders). The UCSF Benioff Children’s Hospital, Oakland, institutional review board reviewed and approved the study. All of the women signed a consent form after thoroughly reviewing the study details with research personnel and after discussing the study with family members and friends. The participants received a stipend of $300 for completing both test days.

### 2.3. Study Day Procedures

On the test days, participants arrived at the CRC between 7:30 AM and 8:30 AM after a ten-hour overnight fast. The women were told to drink at least eight ounces of water prior to coming to the CRC. Upon arrival, the women relaxed in a reclining blood-draw chair and an indwelling catheter was placed in the antecubital vein. After collecting the fasting blood sample, the women consumed one of the two test meals within 20 min. Subsequent blood samples were drawn through the indwelling catheter at 30 min, 1, 2, 3, 4, and 5 h. Prior to each blood draw, participants completed a visual analogue satiety (VAS) questionnaire. At the end of the postprandial study, participants were offered a buffet lunch with individual weighed portions for each food item. Total food consumption was determined from the difference between the weighed amounts of food offered and any uneaten food. 

### 2.4. Test Meals

Since carbohydrate and lipid postprandial metabolism may be influenced by food intake during the past six to eight hours [19], a standard evening meal was provided prior to each study day. The 600-kcal, standard dinner consisted of a frozen ovo-lacto vegetarian entrée along with fruit and a non-caloric beverage. 

The characteristics of the two standardized breakfast test meals are shown in Table 1. Both meals contained 240 g of apple juice and a 65-g bagel. For the almond (nuts) test meal, 60 g (approximately two ounces) of blended almonds was served. For the cream cheese (dairy) test meal, 86 g (approximately three ounces) plus a small amount of supplemental Beneprotein (Nestle Healthcare Nutrition, Minnetonka, MN, USA) and Polycose (Abbott Nutrition, Abbott Park, IL, USA) powders to equalize the amount of carbohydrate and protein in the two meals was served. The nut meal was lower in saturated fat and higher in total fiber. Palatability and acceptability of both test meals was previously tested in non-pregnant volunteers [20].

### 2.5. Body Anthropometrics and Composition

All measurements of body anthropometrics and composition were done at the second study visit. Body weight was measured in undergarments to the nearest 0.1 kg using a standing digital scale (BWB-800, Tanita, Arlington Heights, IL, USA). Height was measured to the nearest 0.1 cm by using a wall-mounted stadiometer (Perspectives Enterprises, Portage, MI, USA). All measures were done in duplicate and averaged. Maternal BMI was calculated as: weight (kg)/height (m)^2^.

Body density was estimated using the BODPOD body composition system (Life Measurement Instruments, Concord, CA, USA), which estimates body volume from the amount of air displaced by a subject’s body when seated in a fiberglass chamber [21]. Body density was measured two hours after the participant had consumed the test meal. Participants wore tight-fitting clothing, a swim cap, nose clip without any jewelry or accessories for the measurement. After voiding, the participants entered the BODPOD chamber, and body volume was measured twice during a five-minute time period and averaged. Thoracic gas volume (TGV) was also measured by having the participant blow into an attached breathing tube while in the chamber. If a valid TGV measurement could not be made, an equation for predicting TGV was used [22]. Body density was calculated from the ratio of body mass (weight) to body volume. A three-compartment model was used to estimate percent body fat and body fat-free mass [23] and the methods are detailed in our previous study [24].

### 2.6. Blood Sampling, Aliquoting, and Analysis

Blood samples were collected from the antecubital vein of recumbent subjects in the fasting state and at 30 min, 1, 2, 3, 4, and 5 h postprandial. Blood samples were separated for serum or plasma via centrifugation and stored at −80 °C until analysis. The collection times and methods for analysis are shown for each endpoint in Table 2.

### 2.7. Satiety Assessment

Immediately before each blood sampling, the participants were given a VAS satiety questionnaire [16]. Satiety was also assessed by measuring the *ad libitum* food intake from a buffet of pre-weighed lunch items. The same buffet menu was fed on both study days.

### 2.8. Statistical Analysis

Power calculation: The glycemic response to the test meals was the primary outcome of interest. In a previous study of seven individuals with type 2 diabetes mellitus [28], the postprandial glucose area under the curve averaged 429 ± 75 mmol/min/L following the control meal and 299 ± 63 mmol/min/L following the almond meal, about a 30% reduction (*p* < 0.04). Since the women in our study did not have diabetes, we predicted that the glycemic response difference between the dairy and nut meals would be smaller, about 80 mmol/min/L rather than 130 mmol/min/L. A sample size of 11 women was needed to detect a difference of 80 mmol/min/L using a two-tailed test when α = 0.05 and Power = 0.80.

Data was transferred to SAS data sets and analyzed using SAS software version 9.3 (SAS Institute, Inc. 2010, Cary, NC, USA). The value of *p* = 0.05 was used as the cut-off level for statistical significance [29,30]. Prior to any analyses, data were checked for outliers. Once the data set was finalized, a descriptive analysis for each time point in each diet group was done to examine means, proportions, measures of variability and correlations.

Both cross-sectional and longitudinal analyses of the data were performed using generalized linear mixed-effects models (GLMM) to test the hypothesis that the metabolic response to the control meal (dairy, cream cheese) differed from the intervention meal (nuts, almonds) over time. The longitudinal models accounted for any within-subject correlations over time and with the two diets. A series of models were run to test the hypothesis that a breakfast meal with nuts differed from dairy in appetite or metabolites of glucose or lipid metabolism. The association between fasting and postprandial lipid profiles was also determined. 

For each fasting state and postprandial biomarker outcome, we first screened for any sequence effects (order of test meal received) while controlling for diet, time postprandial and study visit 1 or 2 in the models. Since no meal sequence effects were observed, sequence was excluded from each model and data from both study visits were used. For data measured only during the fasting state (HMW Adiponectin, hs-CRP, Total-Cholesterol, LDL-cholesterol, HDL-cholesterol, lipoprotein particle size, HgbA1c), the models included diet and study visit. For the postprandial data, each model controlled for baseline values of the biomarker assessed. Furthermore, Tukey’s method of multiple comparisons was used to assess any differences between the dairy and nut meals at each time point. A separate model was used to explore the relationship between potential covariates (lipid profile, pre-pregnancy weight, pre-pregnancy BMI, gestational age, gestational weight gain, and measured body fat) with each of the measured outcomes at all time points in order to determine which covariates could be adjusted for in the model.

For the VAS satiety questionnaire responses, analysis of variance models (ANOVA) and the Wilcoxon signed rank test were used to determine differences in response at each time point. The Wilcoxon signed rank test was also used to determine the breakfast test meal effect on energy consumption from the lunch meal.

## 3. Results

### 3.1. Participant Characteristics

Eighteen (two Asian and sixteen Latina) pregnant women aged 28 ± 5.9 years participated in the study. Their self-reported pre-pregnancy weight averaged 77.6 ± 16.4 kg with a corresponding mean BMI of 31 ± 5.9 kg/m^2^. Estimated gestational weight gain at the first study visit (i.e., 32.6 weeks gestation) averaged 6.3 ± 4.7 kg (Table 3). An additional 1.3 ± 4.0 kg was gained by the second study visit approximately 1 week later bringing their total weight gain to 7.7 ± 4.9 kg at 33 weeks gestation. Total body fat, measured at the second visit, was 41.9 ± 5.5%. The average hemoglobin A1c level, 7.2 ± 0.6%, was slightly elevated compared to what is expected in pregnancy (6–7% [31]), suggesting an underlying insulin resistance. Fasting serum adiponectin levels on visit 1 were also on the lower end (7.3 µg/mL) of the normal range (4–22 µg/mL, for women with BMI >30), which is consistent with insulin resistance [32,33]. Total cholesterol levels, averaged 230.3 ± 32 mg/dL, reflecting the typical increase in serum cholesterol seen in pregnancy [34]. The mean LDL peak diameter was 217.8 Å, with 36% of the participants having a small, dense LDL phenotype B profile.

### 3.2. Metabolic Markers

**Figure 1:**
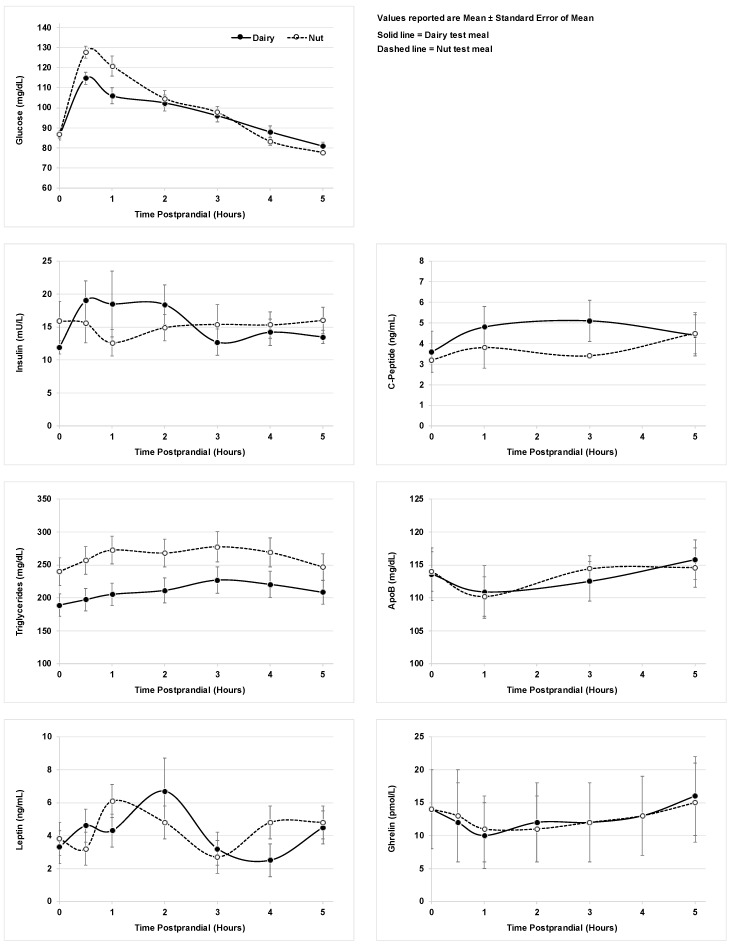
Participant metabolic parameters after consumption of dairy vs. nut test meals.

The response of the metabolic biomarkers to the two test meals over time is shown in Figure 1. None of the metabolites differed with the type of breakfast meal consumed at any of the postprandial time points. Postprandial serum glucose, triglyceride, and ghrelin concentrations changed significantly over the 5 h postprandial period. Serum glucose concentrations declined significantly during the 5 h study period (*p* < 0.0001), while serum triglyceride and ghrelin concentrations significantly increased (*p* = 0.002, *p* = 0.006). Insulin, c-peptide, apolipoprotein B and leptin concentrations did not change significantly during the postprandial period.

### 3.3. Satiety Questionnaires and Buffet Lunch

VAS questionnaires were used to assess hunger following the two meals. The dairy meal satiety response was slightly higher than that for nuts, but the difference was not significant. The satiety response within a meal varied with time (*p* < 0.0001). Compared to the satiety status measured at 0.5 h, the participants reported increased hunger and a lower satiety at 3 h postprandial. When the cream cheese meal was consumed, the women felt that they could eat more than when they consumed the almond meal (*p* < 0.01) at 3 and 4 h postprandial. However, the ad libitum caloric intake at the post-test lunch meal did not significantly differ between the two test meals. Mean total energy consumption was 811 ± 288 kcal following the cream cheese test meal and 787 ± 260 kcal following the almond test meal (*p* = 0.79).

## 4. Discussion

In this study of overweight/obese pregnant women, consuming cream cheese (dairy) or an isocaloric and equal macronutrient amount of almonds (nuts) did not alter postprandial metabolism or subsequent meal intake amount. Since an energy equivalent amount of nuts provides more unsaturated fat and fiber than cream cheese, we hypothesized that the postprandial metabolic glucose and triglyceride response to almonds would be more favorable than the response to cream cheese. Previous test-meal studies in non-pregnant adults have shown an improved postprandial glycemic response when almonds were consumed [17,35]. However, those studies were not isocaloric and had different amounts of fat as the metabolic effects of almonds were compared to white bread. In our study, almond consumption did not shift the postprandial insulin, leptin, or ghrelin response, and, consequently postprandial blood glucose and lipid levels did not change. 

The amount of fiber was 4.5 times higher and the amount of saturated fat 6 times lower in the nut meal compared to the dairy meal. Also, almonds contain higher amounts of certain micronutrients, such as calcium, that have a role in energy metabolism and promoting fat oxidation [36]. Ideally, postprandial studies of fat metabolism are longer than the 5 h used in this study because the metabolic effects of the dietary fat may not be evident for 8 to 10 h [37]. Nevertheless, short-term studies show that replacing saturated fatty acids with polyunsaturated fatty acids, such as those found in nuts, decreases the serum triglyceride postprandial response and markers of inflammation [38]. In our study, the postprandial serum triglyceride levels were unchanged at 5 h after both test meals. 

The response to our satiety questions did not differ between the two test meals, with the exception that more participants felt they could eat more at 3 and 4 h post consumption of the dairy meal compared to the nut meal. Biomarkers of satiety, i.e., leptin and ghrelin, did not differ following each test meal. We may have failed to see a satiety difference between our two meals because our study was powered to detect differences in blood glucose rather than indicators of satiety or because the macronutrient amounts were the same for both test meals.

Although the intake of nuts in our population of pregnant women did not alter metabolic biomarkers compared to the intake of dairy, the addition of healthy fats and fiber (such as those found in almonds) to the diets of high-risk obese pregnant women should be considered. Several randomized, controlled, cross-over trials show that nuts may reduce cardiovascular disease risk by altering inflammation, oxidation, glucoregulation, and circulating cholesterol [39,40,41,42]. Advising overweight/obese women to consume unsaturated fatty acid-rich food items, prior to and during pregnancy may enable them to enter pregnancy in a healthier state and to reduce their risk of metabolic disorders during pregnancy.

## 5. Conclusions

In summary, our data show that consuming dairy or nuts in isocaloric test meals with equivalent amounts of total fat, carbohydrate and protein had a minimal effect on postprandial metabolism as measured by the response of glucose and lipid parameters as well as satiety and subsequent energy intakes in overweight/obese pregnant women. The effect of pregnancy on the postprandial metabolic response may be greater than the impact of the food items consumed.

## Figures and Tables

**Table 1:** Test Meal Macronutrient Characteristics.

Test Meal	Meal Contents	Total Calories	Total Fat (% Energy)	Saturated Fat (% Energy)	Total Protein (% Energy)	Total Carb (% Energy)	Total Fiber
Dairy (Cream Cheese)	65 g bagel 240 g apple juice 86 g cream cheese 9 g Polycose powder 8 g Beneprotein powder	665 kcal	31 g (42%)	18 g (24%)	19 g (12%)	77 g (46%)	2 g
Nut (Almonds)	65 g bagel 240 g apple juice 56.7 g almonds	665 kcal	31 g (42%)	3 g (4%)	19 g (12%)	77 g (46%)	9 g

**Table 2:** Blood Analyses.

Parameter	Sample Collection Times Points (Hours)	Analysis Method	Sample Collected (+ Additives If Any)
Glucose	0, 0.5, 1, 2, 3, 4, 5	Polychemical Clinical Analyzer, MedTest DX (Cortlandt Manor, NY)	Plasma
Triglyceride	0, 0.5, 1, 2, 3, 4, 5	Polychemical Clinical Analyzer, MedTest DX (Cortlandt Manor, NY)	Plasma
Ghrelin	0, 0.5, 1, 2, 3, 4, 5	Multi-array Electrochemiluminescence (Human Ghrelin Prototype Assay), Meso Scale Discovery (Rockville, MD)	Plasma + aprotinin inhibitor
Insulin	0, 0.5, 1, 2, 3, 4, 5	Multi-array Electrochemiluminescence (Human Active GLP-1, Insulin, Glucagon, Leptin Assay Kit), Meso Scale Discovery (Rockville, MD)	Plasma + DPP-IV inhibitor + aprotinin inhibitor
Hemoglobin A1c	0	Enzymatic, Diazyme (Poway, CA)	Whole blood
Leptin	0, 0.5, 1, 2, 3, 4, 5	Multi-array Electrochemiluminescence (Human Active GLP-1, Insulin, Glucagon, Leptin Assay Kit), Meso Scale Discovery (Rockville, MD)	Plasma + DPP-IV inhibitor + aprotinin inhibitor
C-peptide	0, 1, 3, 5	Multi-array Electrochemiluminescence (Human C-Peptide Prototype Assay), Meso Scale Discovery (Rockville, MD)	Plasma
Apolipoprotein B	0, 1, 3, 5	Immunoturbidimetric assay [25,26] (Kamiya Biomedical Company, Seattle, Washington), Liasys 330 automated chemistry analyzer	Serum
High Molecular Weight Adiponectin	0	Multi-array Electrochemiluminescence (Human Adiponectin Assay Kit), Meso Scale Discovery (Rockville, MD)	Plasma
High-sensitivity C-Reactive Protein	0	Multi-array Electrochemiluminescence (C-Reactive Protein Assay), MSD (Rockville, MD)	Plasma
Total-cholesterol	0	Enzymatic end-point measurement (AMS Diagnostics, Brookfield, Connecticut), Liasys 330 automated chemistry analyzer	Plasma
Low Density Lipoprotein-cholesterol	0	Calculated by subtraction of estimated Very Low Density Lipoprotein and measured High Density Lipoprotein cholesterol from the measured total cholesterol and triglyceride in plasma	Plasma
High Density Lipoprotein-cholesterol	0	Enzymatic end-point measurement (AMS Diagnostics, Brookfield, Connecticut), Liasys 330 automated chemistry analyzer	Plasma
Lipid-particle size	0	Ion mobility (IM) analysis, electrospray utilized to create an aerosol of particles that pass through a differential mobility analyzer (DMA) coupled to a particle counter [27]	Plasma

**Table 3:** Participant Characteristics.

Characteristic	Mean ± SD or Percentage
Weeks of gestation	32.6 ± 1.8
Weight, kg	84.6 ± 15.2
Estimated Gestational Weight Gain, kg ^a^	6.3 ± 4.7
Body Fat, %	41.9 ± 5.5
Blood Pressure Systolic Diastolic	104 ± 10.7 63 ± 5.9
Hemoglobin A1c, %	7.2 ± 0.6
Glucose, mg/dL	86.5 ± 7.4
Insulin, mU/L	13.9 ± 7.9
Triglycerides, mg/dL	214.4 ± 79.5
Leptin, ng/mL	3.60 ± 3.2
Ghrelin, pmol/L	14.1 ± 6.2
Adiponectin, ug/mL	7.3 ± 3.6
C-reactive protein, mg/dL	0.2 ± 0.1
Total cholesterol, mg/dL High Density Lipoprotein-cholesterol, mg/dL Low Density Lipoprotein-cholesterol, mg/dL Very Low Density Lipoprotein-cholesterol, mg/dL	230.3 ± 32 74.2 ± 21.3 115.3 ± 34.3 40.8 ± 13.6
Low Density Lipoprotein Peak Diameter (Å)	217.8 ± 5
Apolipoprotein B, mg/dL	114 ± 24.6
Low Density Lipoprotein Phenotype (%)	
A	52.8%
I	11.1%
B	36.1%

^a^ Estimated Gestational Weight Gain at visit 1; an additional 1.3 ± 4 kg of weight was gained between visit 1 and 2 bringing the total to 7.7 ± 4.9 kg at 33 weeks gestation.
